# Advancing engagement methods for trials: the CORE study relational model of engagement for a stepped wedge cluster randomised controlled trial of experience-based co-design for people living with severe mental illnesses

**DOI:** 10.1186/s13063-017-1878-7

**Published:** 2017-04-08

**Authors:** Lauralie Richard, Donella Piper, Wayne Weavell, Rosemary Callander, Rick Iedema, John Furler, David Pierce, Kali Godbee, Jane Gunn, Victoria J. Palmer

**Affiliations:** 1 The Department of General Practice, Melbourne Medical School, Faculty of Medicine, Dentistry and Health Sciences, The University of Melbourne, 200, Berkeley Street, Melbourne, VIC 3052 Australia; 2 The Department of General Practice and Rural Health, Dunedin School of Medicine, University of Otago, Dunedin, New Zealand; 3 School of Health, University of New England, Armidale, Australia; 4 Monash Centre for Health Research and Implementation, Monash University, Clayton, Australia; 5 Department of Rural Health, Faculty of Medicine, Dentistry and Health Sciences, The University of Melbourne, Melbourne, Australia

**Keywords:** Engagement model, Stepped wedge design, Cluster randomised controlled trial, Complex interventions, Experience-based co-design

## Abstract

**Background:**

Engagement is essential in trials research but is rarely embedded across all stages of the research continuum. The development, use, effectiveness and value of engagement in trials research is poorly researched and understood, and models of engagement are rarely informed by theory. This article describes an innovative methodological approach for the development and application of a relational model of engagement in a stepped wedge designed cluster randomised controlled trial (RCT), the CORE study. The purpose of the model is to embed engagement across the continuum of the trial which will test if an experience-based co-design intervention improves psychosocial recovery for people affected by severe mental illness.

**Methods:**

The model was developed in three stages and used a structured iterative approach. A context mapping assessment of trial sites was followed by a literature review on recruitment and retention of hard-to-reach groups in complex interventions and RCTs. Relevant theoretical and philosophical underpinnings were identified by an additional review of literature to inform model development and enactment of engagement activities.

**Results:**

Policy, organisational and service user data combined with evidence from the literature on barriers to recruitment provided contextual information. Four perspectives support the theoretical framework of the relational model of engagement and this is organised around two facets: the relational and continuous. The relational facet is underpinned by relational ethical theories and participatory action research principles. The continuous facet is supported by systems thinking and translation theories. These combine to enact an ethics of engagement and evoke knowledge mobilisation to reach the higher order goals of the model.

**Conclusions:**

Engagement models are invaluable for trials research, but there are opportunities to advance their theoretical development and application, particularly within stepped wedge designed studies where there may be a significant waiting period between enrolment in a study and receipt of an intervention.

## Background

Engagement is an important component of community-based interventions, trial designs and health research broadly. However, relatively few studies report on the engagement of public, patient and/or service user across the research continuum. When engagement methods are reported, the description of the process of engaging with participants is largely focussed on recruitment and retention [1]. Despite having been experimented in a certain number of studies [2–4], transversal engagement—that is, engagement that runs from the research design phase through to the translational phase of the research continuum—is minimal. This has been the case in trials research with some exceptions. Previous systematic reviews have described various aspects of the engagement process for involving patients and services across the continuum in terms of design, execution and translation, but it still remains unclear how to perform this task and for how long, particularly in the context of trial designs [1, 5, 6]. Furthermore, there is a growing consensus that term engagement is conceptually confusing and would benefit from theoretical development [5, 7].

Despite conceptual difficulties, engagement has increased its prominence in health and medical science research [8]. Here it is important to note that the methods of engagement in research are distinguished from patient engagement in direct care and treatment and are different from other community engagement and public participation and involvement (PP&I) methods such as deliberative democracy panels and citizen juries [9, 10]. Engagement in research draws on the principles of choice and shared decision-making that are central to community engagement, patient engagement and PP&I methods, but in its ideal form it expands on these principles. While patient and community engagement and PP&I harness the public and patient as decision-makers and similarly emphasise empowerment through giving voice, engagement in research encompasses user involvement in the design processes, reciprocal exchange of information to inform recruitment strategies from key stakeholders (co-produced strategies), ongoing interactional communication methods over the life of studies and increasing involvement of the subjects of research as data collectors and analysts [1, 11]. These newer forms of engagement disrupt traditional roles of researchers and subjects and ensure that lived experience is a central and valued element of the research endeavour. Engagement in research has certainly advanced beyond the early forms of involving lay persons in the assessment and review of research and service user representation on advisory panels [8, 12–17].

Despite the expanded role of research participants in the design, participation and conduct of studies, a good deal of literature still confines research engagement to ways in which researchers can increase recruitment through innovative methods to encourage research participation, how to conduct knowledge exchange and methods for translation of research findings with potential users, for example, ’the public’ or ’government’ [1, 18]. Getting to the stage where an engagement model is embedded across the trial design should be a goal of future research. To do this, we need to move beyond a perception of engagement for instrumental purposes where there is little to no sustained engagement efforts deployed once participants have enrolled in the research [19]. In other words, engagement should not revolve solely around the system’s agenda (e.g. research goals of improving recruitment and retention) but should also try to incorporate the participant’s agenda [20].

A shift in the way that we think about engagement within trials research and complex interventions is thus needed. Continuous (rather than episodic) and relationally driven approaches to engagement are therefore required within trial designs and complex interventions. These approaches concentrate on the ’web of relationships’ that interconnect researchers, research participants and the public, and act as the ’human infrastructure’ and foundation for meaningful connections to occur in a research context, with the ultimate goal to foster socially and ethically responsible research [21]. Our view is that such approaches are fundamental to ensuring that research is translated beyond the funded life of studies and to generate conditions for interventions to later become embedded within healthcare systems.

For this reason, we have purposively used the term ‘relational’ in our model to signify the shift from instrumental and transactional approaches to viewing engagement through a lens of enduring commitment and outcomes. This, we think, emphasises the human dynamics and dimensions of engagement, which translate into the network of social relationships that structure the engagement process and strategies as well as the roles, responsibilities and levels of engagement and participation available to those who are involved in the research [21]. Thus, ’relational engagement’ stresses the importance of the human and fundamentally interactive nature of engagement in research and promotes socially responsible and protective relationships for the participants involved. It is premised on relational ethical theories whereby the relational world of research participants and stakeholders is paramount, including their personal stories and identities, and efforts are made to understand and acknowledge these features as central in how decisions and actions are shaped. This has particular relevance for research engaging with vulnerable groups such as the participants involved in the CORE study which we explain in full in the following sections.

**Fig. 1:**
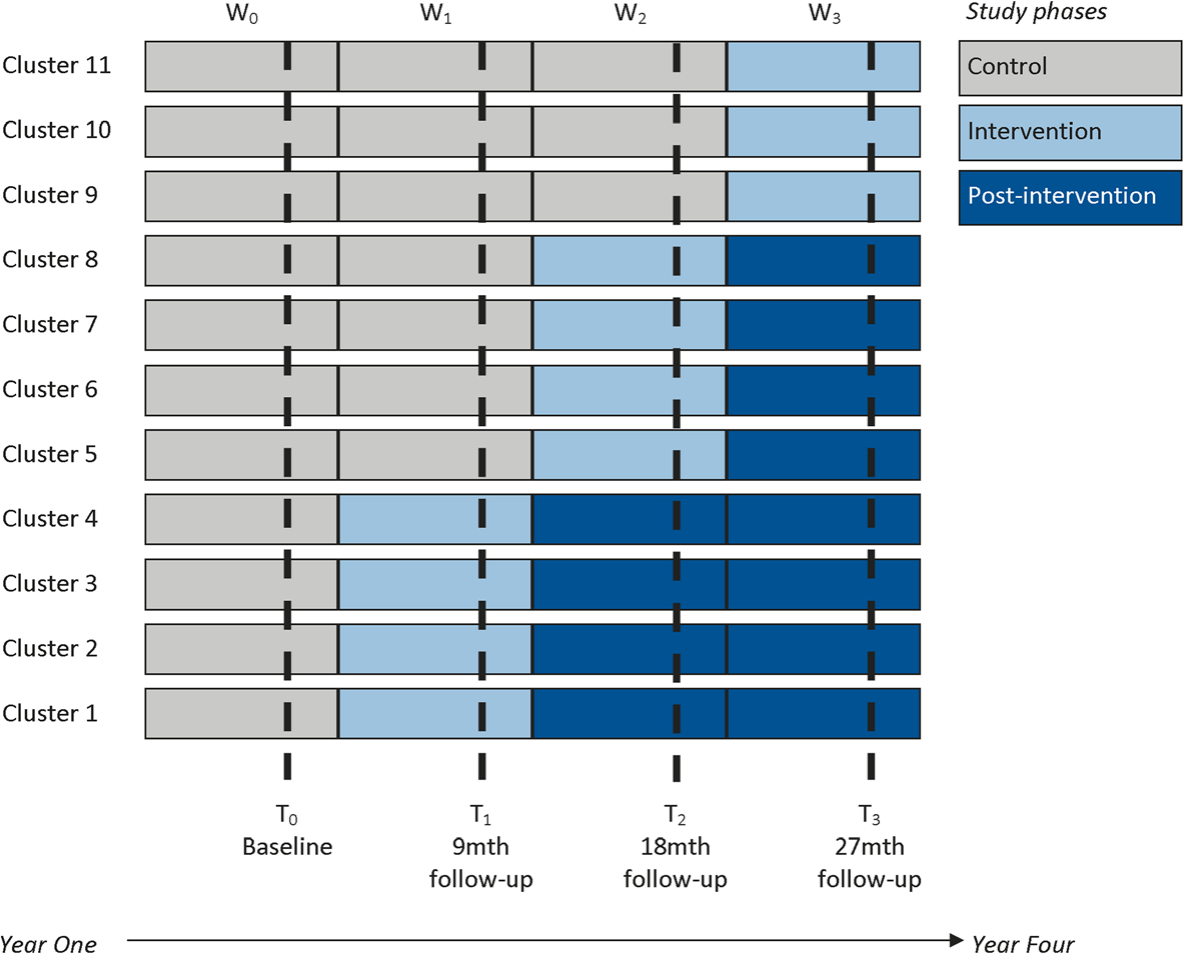
The CORE study stepped wedge cluster randomised controlled trial design

**Fig. 2:**
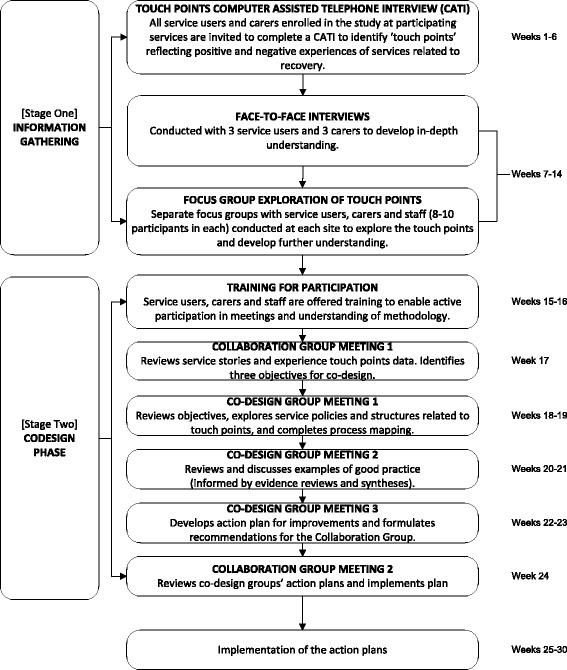
Intervention flow for the CORE study (modified Mental Health Experience Co-design, MH ECO)

In this paper, we outline the development and application of an innovative model for engagement. The model was designed to work across the continuum (design to translation) of the CORE stepped wedge cluster randomised controlled trial (RCT). The CORE study protocol has already been published and the full trial details are summarised in Table 1 [22]. Figure 1 illustrates the stepped wedge study design. In brief, CORE is testing if an experienced-based co-design (EBCD) methodology called Mental Health Experience Co-design (MH ECO) for people living with severe mental illness in Victoria, Australia improves psychosocial recovery outcomes. MH ECO brings together service users (people with severe mental illness), carers and mental health staff to identify things going well in service experiences and the areas for change (detailed in Fig. 2). Following the identification of the areas for improvement, service users, carers and staff participate in a structured and facilitated process to co-develop solutions for implementation in community mental health services. The engagement work inherent to the trial design is coherent with the participative nature of the intervention that is being tested, but the model does not refer to the engagement of the participants within the co-design intervention proper.

**Table 1:** Summary of the CORE study stepped wedge cluster randomised controlled trial protocol

*Context* User engagement in mental health service design is heralded as integral to health systems quality and performance, but does engagement in re-designing services improve health outcomes?
*Objective* To test the effectiveness of engaging service users, carers and staff in community mental health services in an experience-based co-design intervention to improve individual psychosocial recovery, carer well-being, staff attitudes to recovery and the recovery orientation of services.
*Design setting participants* A stepped wedge cluster randomised controlled trial with a nested process evaluation will be conducted over nearly 4 years in Victoria, Australia. 11 teams from four community mental health service providers will be randomly allocated to one of three dates 9 months apart to start the intervention. Data will be collected at baseline and at completion of each intervention wave (9, 18 and 27 months). Participants will be 30 service users, 30 carers and 10 staff working in each cluster (team) of four major mental health service providers.
*Intervention* The intervention is a modified version of Mental Health Experience Co-Design (MH ECO). MH ECO is a two-staged method. Stage 1 involves the identification of positive experiences and the aspects of service experience that could improve. Stage 2 involves smaller groups of service users, carers and staff participating in structured and facilitated meetings to co-develop improvements and action plans for change.
*Outcome measures* The primary outcome is improvement in psycho-social recovery score using the 24-item Revised Recovery Assessment Scale (RAS-R) for service users. Secondary outcomes are improvements to user and carer quality of life and well-being using the shortened 8-item version of the World Health Organisation Quality of Life (WHOQOL) scale (EUROHIS), changes to staff attitudes using the 19-item Staff Attitudes to Recovery Scale (STARS) and recovery orientation of services using the 36-item Recovery Self Assessment Scale (RSA-provider version).
*Analysis* Intervention and usual care periods will be compared using a linear mixed effects model for continuous outcomes and a generalised linear mixed effects model for binary outcomes. Participants will be analysed in the group to which the cluster was assigned at each time point.

The trial design includes a nested process evaluation (NPE) for which the protocol is already published [23]. One of the objectives of the NPE is to test if the relational model of engagement increased recruitment and retention of study participants (comparative with other studies of the same target population) and if the embedded model facilitated knowledge transfer in the trial (using knowledge translation theories). These results will be reported in the process evaluation. The CORE model of relational engagement has been designed to foster meaningful connections with key target groups within CORE, namely service users, carers, frontline staff in community mental health services and management- and executive-level staff of mental health service organisations. As part of this process, careful attention has been given to building connections beyond the key target groups for the trial to include key stakeholders such as government agencies and other non-government organisations for whom the research being undertaken could benefit.

We propose that there are many cluster and individually randomised controlled trials adopting a stepped wedge design which require a similar model of engagement since there may be a significant delay between enrolment and receipt of an intervention due to the nature of this design [24]. As Fig. 1 illustrates, every cluster in the CORE study will ultimately receive the co-design intervention. However, since clusters are randomised to one of three waves 9 months apart, there may be more than 12 months to 1.5 years wait time to receive the intervention [25]. Thus, having mechanisms to continue to keep people engaged in the research processes is essential both for participant motivation and benefit and to ensure adequate power for analyses. Importantly however, engagement of participants should not just be about meeting the end point, for example, retention, as we will demonstrate in the following discussion. We begin by outlining the methodological process undertaken to develop the relational engagement model and present its theoretical underpinnings as part of advancing engagement in trials research.

## Methods

The relational engagement model for the CORE study was designed and developed during the study establishment phase (June 2013–October 2014). The agenda setting and funding proposal was co-developed in conjunction with partner agencies the Victorian Mental Illness Awareness Council (VMIAC) and Tandem, representing Victorian mental health carers, who allocated two researchers to the investigator team. Model development followed a bottom-up, iterative process completed over three stages. The first stage included a context mapping method [26–28] of the participating organisations, trial sites, policy context and geographical areas and, where possible, information about the target populations (service users and carers). The contextual, local characteristics and dynamics of services and some of the service users’ characteristics were assembled into descriptive portraits of each organisation. Data for the portrait development included summary information from face-to-face interviews conducted with senior managers (*n* = 4); service user/peer consultants employed by services (*n* = 2); analysis of key mental health policy, legislation and government reform documents; and summative information from organisational websites about available mental health programs and recovery philosophies articulated by the service organisations. Descriptive portrait data were supplemented with snapshot information of well-being indicators such as population growth and community composition and transport access and health indicators such as smoking rates, health service utilisation and mental well-being available from local government authorities.

Following this contextual review, the second stage involved consulting the literature on barriers to recruitment and retention of hard-to-reach groups in complex interventions and RCTs relevant to the CORE study. From this we determined existing barriers to recruitment which are summarised in Table 2.

**Table 2:** Barriers and challenges to recruitment

Barriers and challenges to recruitment and participation [1, 6, 10, 29–33]	Examples from the literature
Geographical factors	Relocation of participants, transportation difficulties
Illness-related barriers	Fear of relapse as a result of participation, hospitalisation, being medicated, medication change or other treatment issues, severity of illness, early phase of illness, unstable mental state, symptoms of mental illness, acceptance of illness
Level of support	Lack of support to take part in research, ’no one to go with’
Belief in one’s capabilities	Low self-efficacy, self-esteem or confidence, lack of motivation, goals and aspirations
Fear, suspicion and/or distrust of researchers and/or general distrust of research	Fear that research could be harmful or cause excessive worry for the person, concerns about confidentiality
General inconvenience of participating in research	Takes too much time, lengthy process involving transportation and attendance
Stigma of mental illness	Fear of being asked about sensitive subjects, invitation to take part in research exacerbates feelings of being labelled by mental illness
System-level/organisational barriers	Competing academic centres studying the same group or conflicting schedules with other programs, tensions between academic institutions and community centres, relying on referrals from clinicians, professionals’ resistance to patients’ participation
Health literacy and language barriers	Lack of familiarity with complex scientific and medical language, low level of health literacy, language difficulties
Research-specific challenges	High commitment of engagement with participants in research, resource-intensive tasks, recruitment difficulties such as problems in finding/recruiting people capable of and interested in participating

The information from Table 2 was combined with our descriptive portrait data, and the investigator team identified some recruitment strategies and engagement approaches. Meetings were held with staff in the participating services to test out the recruitment ideas which led to the final co-produced strategies﻿ from stage two. The third stage involved identification of knowledge transfer and philosophical/ethical theories where relationships are viewed as constitutive, foundational, transversal and co-constructed. These theories provided the underpinning theoretical framework for the relational model of engagement.

## Results

**Fig. 3:**
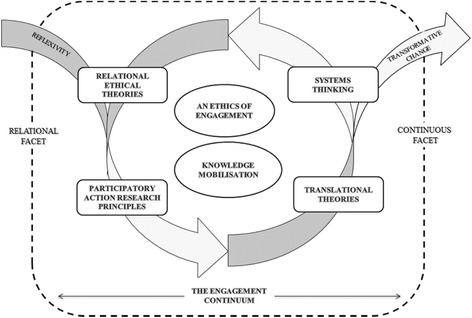
A relational engagement model for the CORE study

After consideration of the contextual knowledge from descriptive portraits and existing literature on barriers to recruitment and retention, we took the four identified theoretical perspectives to build the relational model of engagement. Figure 3 illustrates these perspectives and how they inform the model.

The theoretical framework hinges on two facets: a relational facet and a continuous facet. The relational facet is dependent upon the application of relational ethical theories [34, 35] and the principles of participatory action research [36] to enact an ethics of engagement. The continuous facet uses systems thinking [37] and translational theories [38] to evoke knowledge mobilisation. The theoretical frameworks are explained further below.

### The relational facet

Kenny et al. propose that ‘relational persons develop and deploy their values within the social worlds they inhabit, conditioned by the opportunities and obstacles that shape their lives according to the socially salient features of their embodied lives (for example, their gender, race, class, age, disability status, ethnicity)’ [39]. Adopting a relational ethics approach means that socially salient features need to be considered to assess the webs of relationships that target populations live within to determine how decisions might be made about taking part in a study [40]. In turn, this acknowledges the relationally constituted nature of existence and the importance of identity in fostering stronger, committed and mutually flowing relationships.

**Fig. 4:**
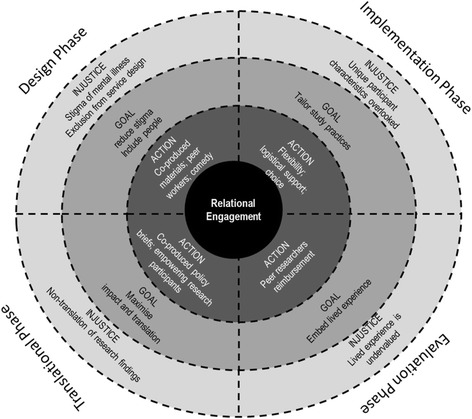
An ethics of engagement for trials research

In Sartre’s notion of existential engagement [35], existence is [viewed as] a social virtue that entails obligations to others. Engagement instructs us to care about the civic conditions through which our identities are shaped and sustained [35]. Engagement rests on three main conditions: *awareness* (being aware of, reflecting on and disclosing injustice); *responsibility* (encouraging others to act and be responsible through disclosure); and *respect* (both for the audience to whom one is disclosing and those experiencing suffering). Combining the relational theoretical approaches with the principles of participatory action research meant that we could invoke a commitment to giving voice, collaboration and empowerment to people who may be on the margins or experience exclusion. The values of the participatory paradigm enabled a closer matching of the needs of all stakeholders and fitted well with local context dynamics, available resources and constraints [41]. The participatory approach provided a space for dialogue and joint action, which fosters an environment conducive to knowledge translation [42]. Taken together, the application of these two theories provided the framework to develop *an ethics of engagement* based on the strategies illustrated in Fig. 4.

Figure 4 highlights some but not all of the strategies for engagement that were developed across the trial continuum. This ethics of engagement is premised on the lived and relational worlds of study participants and the importance of participatory values underpinning research approaches. Within each stage we have illustrated how the Sartrean conditions for engagement were considered by highlighting the injustices (awareness), the goals (taking responsibility) and actions (embodying respect). Our study began from the premise that stigma exists for people living with mental illness and, despite a rhetoric of service user participation, many people remain excluded from meaningful involvement in service planning and re-design. From this awareness we identified the need for a co-design intervention in services that could enable this to occur and work from the basis of empowerment.

From the early design stage our strategies for breaking down stigma and empowering people with lived experience included enlisting people with lived experience in the development of recruitment postcards by using their artwork to capture key messages for participation. We returned to staff within services with recruitment strategy ideas and refined approaches based on their feedback and input. This aspect encouraged staff to buy-in into the study aims and goals and laid the foundation for ensuring their support for recruitment phases. In addition the team sought to disrupt stigma and views of people with lived experience of mental illness by incorporating the Stand Up for Mental Health comedians into study information and recruitment days. We also offered annual engagement events based on comedy and public lectures on new initiatives in reading and writing for well-being that staff in the services were invited to attend. Flexible participation options were embedded into the trial design so that participants could complete one-off surveys or longitudinal follow-up depending on remaining with a service or their ability to participate depending on wellness. We trained peer workers recruited from our partner peak agency VMIAC to be research support workers on the recruitment and information days so that people could talk about the study aims and the requirements for participation in a supportive environment. Where possible, we did recruit people with lived experience to complete telephone interviews with participants, and where it was not possible, we provided training to research staff to foster shared understanding of experience and empathic approaches in data collection. A proportion of funds was allocated to reimbursing participants for their participation in the intervention and to support travel to and from meetings to ensure the best possible opportunities for participation. We had a dedicated logistical coordinator who liaised with service users and carers to provide information about venues, attendance and what to expect during the intervention. Partner agencies and researchers with lived experience delivered the intervention with support from the trained university research team, and they participate in the development of co-produced policy briefs to ensure translation of research findings into practices.

### The continuous facet

Developing a theoretically informed engagement model that can enact an ethics of engagement is essential for creating the conditions for knowledge translation to take place [43–45]. The theory-based model of translation practices (Clavier et al. [38]) was identified as important for structuring some of the engagement activities within the continuous facet further. Clavier et al. [38] suggest that there are three essential translational practices: cognitive, strategic and logistic practices. The cognitive practices refer to the need to circulate knowledge. Here we made efforts to raise awareness amongst partners about the study through face-to-face meetings, study updates, community reports and a dedicated study blog. Specific goals of these practices were to build a common understanding of the research program and study design and to engage people in a way that assisted in developing the research questions. The strategic practices refer to the activities, tools and competencies used to raise and maintain interest of research participants across the lifespan of the study. They aim to facilitate the research process and balance power relationships amongst partners (valuing one’s own interests while also promoting others’, balancing interests for shared power). Here we enlisted staff in the recruitment process and co-developed recruitment strategies with them; we also identified an ambassador in each site to be responsible for sharing study updates and coordinating between the research team and the sites on the ground. Logistic practices correspond to the coordination tasks that create the actual conditions for the engagement. They generally operate with the specific goals of ensuring effective trial management, maintaining partnerships over time, enhancing the recruitment process and facilitating implementation of the research in local settings. Our deployment of a logistical coordinator was part of the endeavour to ensure that effective trial management occurred.

These translational practices combined with systems thinking also allowed for in-depth consideration of the linkages, relationships and interactions amongst the elements that comprise a complex system—that is, one that self-organises, adapts and evolves [46]. The system in which the research is structured, implemented and transformed is referred to here as complex: an interconnected network of actors (experiences, knowledge and values), activities, intentions and projects, as well as environments in perpetual transformation [47]. Systems thinking encourages multifaceted approaches to engagement that are well integrated in local context, involving sustained action and engagement across multiple levels, from the early stages of the research until the knowledge translation phase [37]. It promotes the dynamic engagement of multiple stakeholders and aims to inspire system-wide learning, planning and evaluation. It emphasises the synergies between people-intervention-context and research-practice which are essential to better understand changes that occur over time and emerging outcomes along the way. The engagement strategies we developed sought to grasp the full spectrum of factors at play (e.g. structural, technological, political, cultural, educational, emotional, ethical) [48]. Systems and translational theories were used to evoke *knowledge mobilisation* whereby knowledge was mobilised in a dynamic flow between researchers, participants and the wider community. Table 3 details where the engagement strategies fit within the translational practices. The higher order goal was for the study to become an active process that not only generated data and evidence but contributed to social change.

**Table 3:** Engagement strategies for knowledge mobilisation

Clavier’s three knowledge translation practices	Examples of engagement practices at different stages of research
	Preparatory/pre-randomisation phase: Agenda setting, co-development of the research proposal, prioritisation of the research activities, funding
	Execution phase: Study design and procedures, study recruitment, data collection and data analysis
	Translation phase: Dissemination, implementation and reporting
Logistic Organising and communicating with partners to foster conditions for knowledge translation	From Preparatory/pre-randomisation phase to Translation phase:
	- Regular phone calls to key staff (once a month) - Convening meetings, writing meeting minutes - Managing timetables and deadlines - Organising events and setting up mechanisms for securing partners’ participation - Using a structured approach for communicating with partners through contact logs to keep track of actions, decisions and changes as they occur
Strategic Raise and maintaining partner interest, facilitation of a participatory research process	Preparatory/pre-randomisation phase:
	- Communication of the CORE project goals, design and processes via meetings and research presentations at each site (preparing services to be involved in research) - Clarification of the expectations and identification of the challenges and limits of the research, as well as roles and responsibilities amongst partners
	Execution phase:
	- Study blog for staff and researchers to visit and remain up-to-date with research activities - Tri-annual study newsletters to service users and carers and staff - User-designed posters and postcards for the study located in the wider community (e.g. libraries, community centres, supported residential services, prevention and recovery services)
	From Preparatory/pre-randomisation phase to Translation phase:
	- Site visits every 6 months to talk with teams about the study developments and progress - Recognition of the different agendas, timeframes and professional cultures - Organisation of small and large events to tighten collaborative relationships (regular engagement events throughout the study offered to service users, carers and staff) - Establishment of early communication processes - Regular presence of researchers to decrease feelings of uncertainty towards the research team and build confidence for everyone
Cognitive Developing a shared vision and phrasing of the research	Preparatory/pre-randomisation phase:
	- Partner involvement in the writing of research proposal and setting the agenda of the study - Study information days including verbal education sessions about the project — meet the research team and complete surveys face to face if preferred
	Execution phase:
	- Using trained peer workers for support and a short comedy routine delivered by WISE Employment Stand Up for Mental Health comedians to reduce stigma around mental health and embed lived experience perspectives - Staff distribution of study postcards to potential participants during regular clinical contacts
	From Preparatory/pre-randomisation phase to Translation phase:
	- Creating opportunities for dialogue on the respective contents of the research and mental health services at each site - Engagement events to combat community stigma around mental illness and foster positive views on psychosocial recovery

## Discussion

It is clear that the concept of engagement is important in community-based RCTs, complex interventions and health research. The prominence of patient and public involvement in research now means that it is essential that we develop strategies that enable both the means and the ends of engagement to avoid transactional approaches. There is a need for commitment to higher order goals beyond meeting instrumental needs of recruitment, and clear social change outcomes ought to be embedded within research studies and be a part of the rationale for the engagement process.

Our relational model of engagement illustrates the importance of considering the relational world of participants in research studies and highlights how Sartre’s concept of existential engagement (awareness, responsibility and respect) can be used to orient researchers toward achieving this goal. For trials research the development of theory-informed engagement models at early developmental stages will mean that engagement goes beyond recruitment [49]. There may even be a greater uptake and translation of findings of research studies as a result of more embedded transversal engagement models.

Evidence suggests that we are still striving to find ways of engaging ’well’ with research participants, especially when it involves vulnerable and hard-to-reach groups. In line with South and Phillips, we agree that there is a need to conceptualise and understand engagement in public health improvements and build an evidence base regarding the links with health outcomes in communities [9]. Our model sheds light on two crucial elements for developing engagement models. The first one relates to the importance of using a structured and systemic approach of engagement that spans the continuum of trials to consider the ongoing engagement strategies. As previously mentioned, this has particular relevance for the stepped wedge cluster randomised controlled design, where participants may face a significant waiting period between enrolment at baseline and the receipt of an intervention at different time points. The second one highlights the need for appropriate theories for building models. In our view, these must entail relational, ethical and community-focussed theories.

## Conclusions

Engagement models are essential for research. Such models can help to uncover the synergies and interconnections that occur between participants, context and research, and more importantly to build the foundations for translation beyond the study. Further efforts should be dedicated to design models of engagement in trial development stages and to monitor and document implementation and the outcomes as part of the process evaluation framework [23]. This will contribute to building a better evidence base that moves engagement out of the ‘nice and fluffy’ domains and re-positions it as an essential component of research practice and process [9]. The complexity of engagement and articulating the importance of these relational dimensions will be a challenge in the context of systematic procedural-driven trial methodologies. Ensuring that engagement is coupled with ethical goals and aspirations is an additional challenge, but one that can be met.

### Trial status

The CORE study is registered as a trial with the Australian and New Zealand Clinical Trial Registry, trial ID: ACTRN12614000457640, registration title: The CORE Study: a stepped wedge cluster randomised controlled trial to test a co-design technique to optimise psychosocial recovery outcomes for people affected by mental illness in the community mental health setting. The date registered was 01 May 2014; the start date was 30 June 2014; enrolment of participants was 01 October 2014.
